# A Video Sequence Face Expression Recognition Method Based on Squeeze-and-Excitation and 3DPCA Network

**DOI:** 10.3390/s23020823

**Published:** 2023-01-11

**Authors:** Chang Li, Chenglin Wen, Yiting Qiu

**Affiliations:** 1 School of Automation, Guangdong University of Petrochemical Technology, Maoming 525000, China; 2 School of Automation, Hangzhou Dianzi University, Hangzhou 310018, China

**Keywords:** expression recognition, Squeeze-and-Excitation network, 3DPCANet

## Abstract

Expression recognition is a very important direction for computers to understand human emotions and human-computer interaction. However, for 3D data such as video sequences, the complex structure of traditional convolutional neural networks, which stretch the input 3D data into vectors, not only leads to a dimensional explosion, but also fails to retain structural information in 3D space, simultaneously leading to an increase in computational cost and a lower accuracy rate of expression recognition. This paper proposes a video sequence face expression recognition method based on Squeeze-and-Excitation and 3DPCA Network (SE-3DPCANet). The introduction of a 3DPCA algorithm in the convolution layer directly constructs tensor convolution kernels to extract the dynamic expression features of video sequences from the spatial and temporal dimensions, without weighting the convolution kernels of adjacent frames by shared weights. Squeeze-and-Excitation Network is introduced in the feature encoding layer, to automatically learn the weights of local channel features in the tensor features, thus increasing the representation capability of the model and further improving recognition accuracy. The proposed method is validated on three video face expression datasets. Comparisons were made with other common expression recognition methods, achieving higher recognition rates while significantly reducing the time required for training.

## 1. Introduction

With the rapid development of the internet, the visual information that can be seen in everyday life is taking on an increasingly rich form. Compared to traditional 2D static images, 3D image sequences contain more information, especially temporal information, such as behavioral actions. Facial information is one of the most common sources of information in everyday life, and the visual information it conveys is of great importance [1,2]. In recent years, facial expression recognition has gradually emerged in the field of face recognition and can be applied to psychological research, emotional computing, intelligent interaction, and the medical industry, playing an important role in maintaining social stability and promoting the development of society [3,4,5,6,7,8,9,10,11]. The difference between expression recognition, which is the study of individual differences between different faces, and face recognition, which interferes with information when expressions change; expression recognition is the study of the commonality of facial expressions, and the features extracted reflect the differences between faces in different expression patterns, and the differences in individual faces are interfering information. As a human face moves, there is a dynamic process of change, and its time-domain information has a critical impact on expression recognition.

Traditional research methods can be divided into two categories: feature extraction based on static images and feature extraction of video sequences. Among these, the static images-based feature extraction methods can be subdivided into holistic and local methods, as the static images of facial expressions visualize the changes in facial shape and texture produced by the movement of the muscles of the face as expressions occur. As a whole, this apparent deformation of the facial organs can have an impact on the global information of the face image, hence the emergence of facial expression recognition algorithms that consider expression features from a holistic perspective, such as principal component analysis (PCA), independent component analysis (ICA) and linear discriminant analysis (LDA) [12,13,14,15,16,17,18,19,20]. Yet, there are not only overall variations in facial expressions, but also local variations. The information contained in the local deformation of facial muscles, such as texture and folds, helps to determine the properties of expressions accurately. The classical methods of the local method are the Gabor wavelet [21,22,23,24,25,26] and the Local Binary Pattern (LBP) [27,28,29,30,31,32]. The literature [33] proposes a lightweight face classification method that uses polynomial and smith fuzzy sets set to obtain a new composite fuzzy space to extract different features of images and improve the recognition ability. Research [34] proposes a new face recognition architecture based on the fuzzy discrete wavelet transform (DWT) and two novel local graph descriptors. The proposed new graph-based image descriptors are used to extract salient features for face recognition, and the DWT-fuzzy set-based domain (FWD) method, created by DWT and fuzzy set theory, improves face recognition. As moving images reflect the process by which facial expressions occur, the expression characteristics of moving images are mainly characterized by the continuous deformation of the face and the movement of muscles in different areas of the face. Current dynamic image-based feature extraction methods are mainly divided into the optical flow, model, and geometric methods [35,36,37,38,39,40]. As a human face moves, there is a dynamic process of change, and its time-domain information has a critical impact on expression recognition. Therefore, the facial expression recognition method oriented to video image sequences can better extract the essential change features of expressions and further improve the accuracy of facial expression recognition. With the rise of deep learning, traditional manually designed feature extraction methods have been replaced by deep neural networks. The structure of such networks is often based on the extraction of expression features through several convolutional layers, followed by a non-linear transformation through a fully connected layer, and a Softmax transformation to obtain the probability distribution of samples belonging to each classification. This end-to-end training approach eliminates the tedium of manual feature extraction and, in practice, achieves better recognition results than traditional methods. For 3D data such as video sequences, most models built by convolutional neural networks cannot be processed efficiently. Stretching the input 3D data directly into vectors would not only lead to a dimensional explosion, but also fail to preserve the structural information of the 3D space. The three-dimensional convolutional neural network (3D-CNN) proposed in the literature [41] expands the dimensionality to three-dimensional space, using the same convolutional kernel to perform convolutional operations on three consecutive frames, which can effectively extract spatial and temporal feature information in videos. In the literature [42,43,44], a Long Short-Term Memory network (LSTM) was introduced and cascaded with convolutional neural networks such as CNN, ResNet, and VGG-16, respectively. LSTM has significant advantages in modeling temporal relationships and therefore achieved high recognition accuracy. However, all of these algorithms rely on large training samples and have large parameter computations.

In the field of image recognition, especially in the field based on deep learning, the training speed of the model and the recognition accuracy of the model are usually difficult to be achieve at the same time. Deep learning models are often complex in structure and the number of samples available for training has proliferated with digitalization; once new samples or labels are added, the entire model has to be trained all over again. Such low timeliness often does not meet the actual demand. Therefore, how to reduce the time required for model training while ensuring model accuracy as much as possible is a widespread concern among scholars in the field of deep learning today. This paper proposes a video sequence face expression recognition method based on Squeeze-and-Excitation and 3DPCA Network (SE-3DPCANet). 3DPCANet is a generalization of the lightweight convolutional neural network PCANet in 3D space. Thanks to the better feature extraction capability and lower computational complexity of PCA, 3DPCANet has a faster training speed compared to general deep networks and does not require a large number of training samples. 3DPCANet takes expression image sequences as input samples and introduces the 3DPCA algorithm in the convolution layer to directly construct tensor convolution kernels without weighting the convolution kernels of adjacent frames by shared weights, to extract dynamic expression features of video sequences from spatial and temporal dimensions. Then, to address the redundancy of local channel features, a Squeeze-and-Excitation Network (SENet) is introduced in the feature encoding layer, to automatically learn the weights of local channel features in the tensor features, thus increasing the representation capability of the model and further improving the recognition accuracy.

The main contributions are as follows:

(1) Since the complex information and great data volume of the facial expression data of video sequences and the extremely high training time cost using deep neural networks, this paper proposes use of the lightweight convolutional neural network 3DPCANet for feature extraction of the facial expression data of video sequences. Benefiting from the excellent feature extraction capability, low computational complexity, and simple mathematical mechanism of 3DPCANet, the facial expression features extracted by 3DPCANet greatly reduce the redundancy of the original information while retaining the key information.

(2) In response to the redundancy of local channels, this paper introduces SENet in the feature encoding layer to automatically learn the weights of local channel features in tensor features. The proposed method increases the representation capability of the model and, in combination with 3DPCANet, greatly reduces the time cost of model training while ensuring the accuracy of the model as much as possible. The proposed method is validated on three datasets, compared with a variety of mainstream deep learning models.

The rest of the paper is organized as follows. Section 2 explains tensor representation and arithmetic methods, and a short introduction to 3DPCA and SENet. A video sequence face expression recognition method based on SE-3DPCANet is proposed for expression classification in Section 3. We use three video face expression datasets in Section 4 to validate the superiority of the proposed method for face expression video sequences.

## 2. Preliminaries

### 2.1. Tensor Representations and Operations

This section begins with an introduction to the representations of tensors and the associated operations. Assume that the *N*th-order tensor can be expressed as $X\in R^{I_{1}\times I_{2}\times \ldots \times I_{N}}$.

#### 2.1.1. n-Mode Product

Define the n-mode product of the tensor X and the matrix $U\in R^{J\times I_{n}}$ as $\left(X\times_{n}U\right)\in R^{I_{1}\times \cdots \times I_{n-1}\times J\times I_{n+1}\times \cdots \times I_{N}}$, then its elements can be expressed as:

(1) $${\left(X\times_{n}U\right)}_{i_{1},\cdots ,i_{n-1},j,i_{n+1},\cdots ,i_{N}}=\sum_{i_{n}=1}^{I_{n}}x_{i_{1},i_{2},\cdots ,i_{N}}u_{j,i_{n}}$$

where $i_{n}$ denotes the *N*th order index of the tensor. If $J<I_{n}$, the n-mode product of a multidimensional tensor and a two-dimensional matrix can be dimensionally reduced, i.e., a higher-order tensor is mapped to a lower-order tensor space. In particular, the projection of the tensor to the real numbers can be achieved when the matrix $U\in R^{J\times I_{n}}$ is replaced by the vector $v\in R^{I_{n}}$.

#### 2.1.2. Inner Product

The inner product of two *N*th-order tensors $X,Y\in R^{I_{1}\times I_{2}\times \cdots \times I_{N}}$ of the same size can be expressed as:

(2) $$\langle X,Y\rangle =\sum_{i_{1}=1}^{I_{1}}\sum_{i_{2}=1}^{I_{2}}\cdots \sum_{i_{N}=1}^{I_{N}}x_{i_{1},i_{2},\cdots ,i_{N}}y_{i_{1},i_{2},\cdots ,i_{N}}$$

Accordingly, its Frobenius norm is defined as:

(3) $${\left\|X\right\|}_{F}=\sqrt{\langle X,X\rangle }$$

#### 2.1.3. Outer Product

Define the outer product of an *N*th-order tensor $X\in R^{I_{1}\times I_{2}\times \cdots \times I_{N}}$ and an *M*th-order tensor $Z\in R^{J_{1}\times J_{2}\times \cdots \times J_{M}}$ as:

(4) $$W=X\circ Z\in R^{I_{1}\times \cdots \times I_{N}\times J_{1}\times \cdots \times J_{M}}$$

That is, the order of the tensor W is the sum of the orders of the two tensors.

#### 2.1.4. Kronecker Product

The Kronecker product is the operation defined on the two matrices $A\in R^{I\times J},B\in R^{K\times L}$, i.e.,:

(5) $$A⊗B=\left[\begin{array}{cccc} a_{11}B & a_{12}B & \cdots & a_{1J}B\\ a_{21}B & a_{22}B & \cdots & a_{2J}B\\ \vdots & \vdots & \ddots & \vdots \\ a_{I1}B & a_{I2}B & \cdots & a_{IJ}B \end{array}\right]\in R^{IK\times JL}$$

### 2.2. Three-Dimensional Principal Component Analysis (3DPCA)

Wang et al. [45] established a fast 3DPCA algorithm, which effectively solved the problem encountered when the feature information contained in each image is calculated separately in lung CT image lesion detection, which results in the correlation between individual scan layers will be ignored, thus affecting the detection accuracy. Inspired by 3DPCA, to extract the dynamic features of video face expression sequences and solve the problem of missing dynamic information representation of 2D images, the expression information in time and space dimensions can be represented as a third-order tensor, and the 3DPCA algorithm can be used for feature extraction, and the specific algorithm process is defined as follows:

Assuming a three-dimensional sequence $X\in R^{I_{1}\times I_{2}\times I_{3}}$, where $I_{1}\times I_{2}$ denotes the spatial dimension of each video image frame and $I_{3}$ denotes the temporal dimension of the sequence. The average tensor of this 3D sequence is first calculated:

(6) $$\bar{X}=\frac{1}{I_{3}}\sum_{i=1}^{I_{3}}X_{i}$$

Define the 3D covariance matrix of the sample:

(7) $$\Phi_{\left(3\right)}=\left(X-\bar{X}\right){\left(X-\bar{X}\right)}^{T}$$

Equation (7) can then be transformed into a singular value decomposition (SVD) problem on the $A=X-\bar{X}$. However, the general SVD cannot effectively handle three-dimensional tensors. Therefore, the Higher Order tensor Singular Value Decomposition (HOSVD) is used to compute the singular value decomposition of the tensor A in three-dimensional space [46].

**Figure 1:**
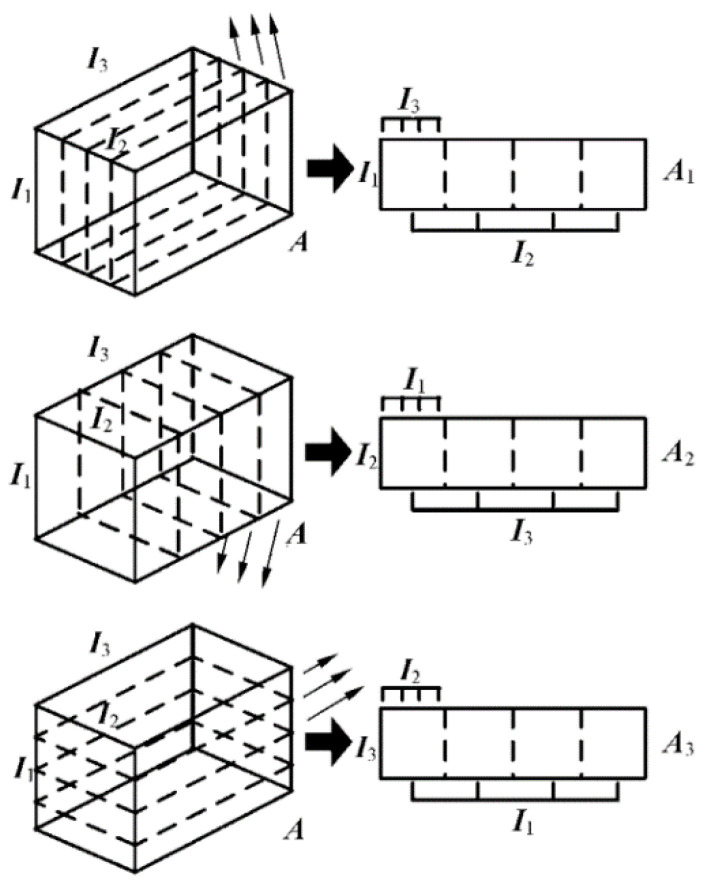
Two-dimensional matrix expansion of a three-dimensional tensor. (The thinner arrow on the left side of the figure represents the direction of tensor expansion).

Firstly, the third-order tensor $A\in R^{I_{1}\times I_{2}\times I_{3}}$ is defined to be expendable, represented as three two-dimensional matrices, $A_{1},A_{2},A_{3}$, corresponding to the three directions in the 3D video sequence, as shown in Figure 1.

As can be seen from Figure 1, slicing and tiling $I_{1}\times I_{3}$ as two-dimensional matrix along the direction of $I_{2}$ yields $A_{1}\in R^{I_{1}\times I_{2}I_{3}}$; slicing and tiling $I_{1}\times I_{2}$ as two-dimensional matrix along the direction of $I_{3}$ yields $A_{2}\in R^{I_{2}\times I_{1}I_{3}}$; similarly, slicing and tiling $I_{2}\times I_{3}$ as a two-dimensional matrix along the direction of $I_{1}$ yields $A_{3}\in R^{I_{3}\times I_{1}I_{2}}$.

Then, the two-dimensional matrix singular value decomposition of $A_{1},A_{2},A_{3}$, respectively, can yield the corresponding unitary matrix $U^{1}\in R^{I_{1}\times I_{1}},U^{2}\in R^{I_{2}\times I_{2}},U^{3}\in R^{I_{3}\times I_{3}}$, and all of these are orthogonal. Then, according to the Tucker decomposition of the tensor, the third-order tensor A can be decomposed into the following product form:

(8) $$A=S\times_{1}U^{1}\times_{2}U^{2}\times_{3}U^{3}$$

where $S\in R^{I_{1}\times I_{2}\times I_{3}}$ is called the kernel tensor. The matrixed solution procedure for the third order SVD can be obtained from Equation (8) and the higher order tensor expansion is as follows:

(9) $$\begin{array}{l} A_{1}=U^{1}\cdot S_{1}\cdot {\left(U^{2}⊗U^{3}\right)}^{T}\\ A_{2}=U^{2}\cdot S_{2}\cdot {\left(U^{3}⊗U^{1}\right)}^{T}\\ A_{3}=U^{3}\cdot S_{3}\cdot {\left(U^{1}⊗U^{2}\right)}^{T} \end{array}$$

where $S_{n}$ is the *n*-th expansion of the kernel tensor S and the symbol ⊗ denotes the Kronecker product. If any two sub-tensor $S_{n_{i}=\alpha },S_{n_{i}=\beta }(\alpha \neq \beta )$ of $S_{n}$ are orthogonal to each other, then the eigenvalues $\lambda_{i}^{n}=||S_{n_{i}=i}|{|}_{F}$, $U_{i}^{n}$ is the corresponding eigenvector. Therefore, we can obtain the main eigenvalues by calculating the cumulative contribution, and then ignore the non-principal components to obtain the new eigen third-order tensor $\hat{S}\in R^{J_{1}\times J_{2}\times J_{3}}$, thus extracting the main feature space $\hat{A}\in R^{J_{1}\times J_{2}\times J_{3}}$ of the 3D video image sequence.

### 2.3. Squeeze-and-Excitation Net (SENet)

The attention mechanism can be seen as a generic pooling method for adaptively assigning weights to inputs and has begun to be used extensively in convolutional neural networks in recent years, providing a huge performance boost in many tasks. Convolution operations can fuse spatial and channel features, and most research has focused on optimizing models for spatial features. The channel attention mechanism, on the other hand, addresses inter-channel relationships and proposes a new Squeeze-and-Excitation Net (SENet). Each feature of the convolution output can be seen as a channel, and the core of this lies in the fact that a constant weight can be predicted for each convolution output feature channel by automatic learning [47].

**Figure 2:**
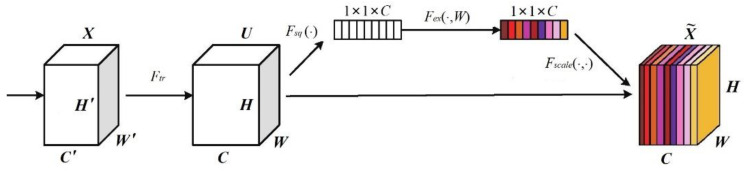
Basic structure of SENet.

The basic structure of the SENet is shown in Figure 2.

Assuming that the convolution outputs *C* H×W-dimensional feature maps, which are considered as *C* channels, we get $U=\{u_{1},u_{2},\cdots ,u_{c}\}\in R^{H\times W\times C}$, then enter the SENet module. The first Squeeze step is performed as follows:

(10) $$z_{c}=F_{sq}\left(u_{c}\right)=\frac{1}{H\times W}\sum_{i=1}^{H}\sum_{j=1}^{W}u_{c}\left(i,j\right)$$

That is, the feature map of channel *C*, dimension H×W, is compressed into a feature map of channel *C*, dimension 1×1, utilizing a global averaging pooling operation, and gets a feature descriptor $Z\in R^{1\times 1\times C}$ that represents global information.

The second step then performs the Excitation operation as follows:

(11) $$S=F_{ex}\left(Z,W\right)=\sigma \left(g\left(z,W\right)\right)=\sigma \left(W_{2}\delta \left(W_{1}Z\right)\right)$$

Equation (11) specifically contains two fully connected layers, and the first full connection is performed on the result *Z* (which can be considered as a *C*-dimensional vector) obtained after global pooling to obtain a C/r-dimensional vector with *ReLU* activation. A full concatenation of the activation results was then performed, turning the C/r-dimensional vector back into a *C*-dimensional vector and performing a *Sigmoid* activation (mapping the weights to between 0 and 1), resulting in a weight matrix.

Finally, the weights of the learned channels are multiplied by the initial features to output the attention-weighted feature map:

(12) $${\tilde{x}}_{c}=F_{scale}\left(u_{c},s_{c}\right)=s_{c}\cdot u_{c}$$

**Figure 3:**
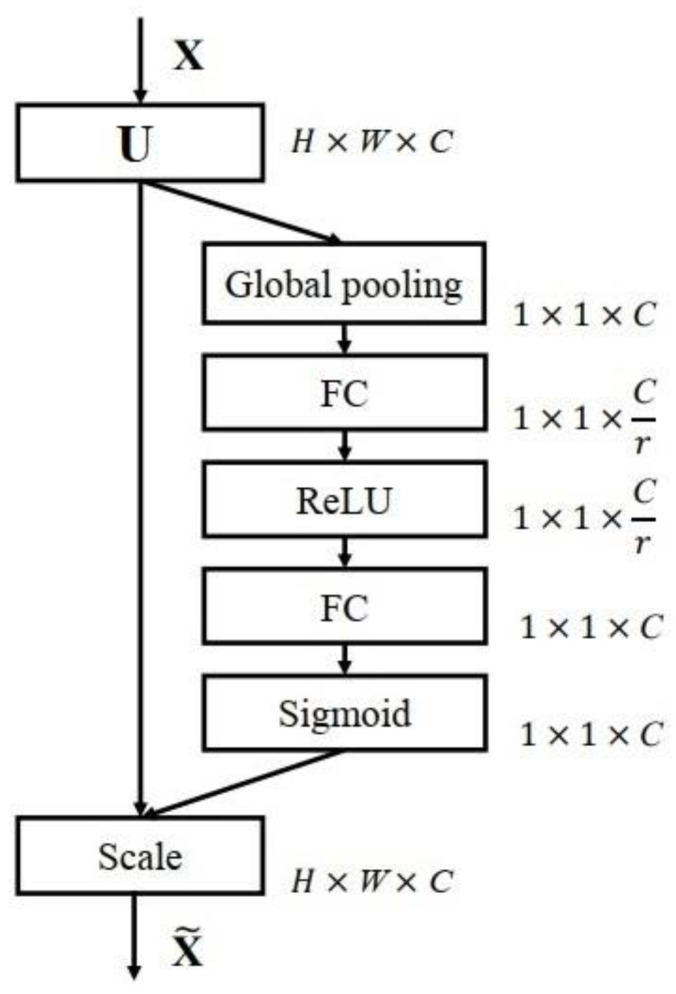
The SENet algorithm process.

The attention-weighted corrected feature map allows for the retention of valuable features and the elimination of less valuable ones. SENet is generic and can therefore be added to existing network architectures, with only a small increase in computational consumption, but with a significant increase in network performance. The overall operation of the SENet is shown in Figure 3.

## 3. A Video Sequence Face Expression Recognition Method Based on SE-3DPCANet

**Figure 4:**
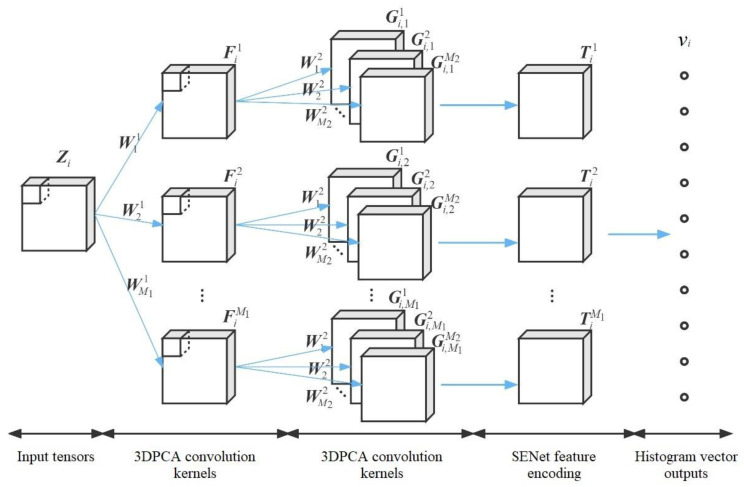
Schematic diagram of the SE-3DPCANet structure.

In this paper, we propose a video sequence face expression recognition method based on SE-3DPCANet. As shown in Figure 4, the model input samples are video expression sequences, and the dynamic expression features are extracted in the convolutional layer using 3DPCA convolutional kernel; for the redundancy of local channel features, SENet is introduced in the feature encoding layer, to automatically learn the weights of local features in the tensor features.

### 3.1. Two-Order Convolutional Layers Based on 3DPCA

Suppose we take a video sequence of *N* sets of facial expressions ${\left\{Z_{i}\right\}}_{i=1}^{N}\in R^{a\times b\times t}$, the size of each image frame in a single expression action sequence $Z_{i}$ is a×b, and the number of video frames is *t*, which is used as the sample input tensors.

Let the tensor sampling block size be $k_{1}\times k_{2}\times k_{3}$, for the *i*th sample $Z_{i}$ can slide sampling to get the tensor set $A_{i}^{1}={\left\{A_{i,j}^{1}\right\}}_{j=1}^{abt}$, where $A_{i,j}^{1}$ denotes the *j*th tensor sampling block of sample $Z_{i}$ in the first convolutional layer. Repeating the sliding sampling operation for *N* sets of samples yields $A^{1}={\left\{A_{i}^{1}\right\}}_{i=1}^{N}$. Then, 3DPCA is performed on $A^{1}$. In all three SVDs, the first $M_{1}$ eigenvectors correspond to the largest eigenvalues, thus obtaining three 2D eigenmatrices:

(13) $$\left\{U_{1}^{1},U_{2}^{1},U_{3}^{1}\right\}=3dpca\left(A^{1}\right)$$

Let $U^{1}={\left\{U_{n}^{1}\in R^{k_{n}\times M_{1}}\right\}}_{n=1}^{3}$, and then use the outer product of the tensor, $U^{1}$ is tensed into $M_{1}$ third-order eigentensor, i.e., the first layer of convolution kernel:

(14) $$W_{l}^{1}=u_{1,l}^{1}\circ u_{2,l}^{1}\circ u_{3,l}^{1}$$

where $W_{l}^{1}\in R^{k_{1}\times k_{2}\times k_{3}}$, $u_{n,l}^{1}\in R^{k_{n}}$, $l=1,2,\cdots ,M_{1}$. Then, sample $Z_{i}$ is convoluted with $M_{1}$ convolution kernels for tensor convolution:

(15) $$F_{i,\;l}^{1}=Z_{i}*W_{l}^{1}$$

Then sample $Z_{i}$ can output $M_{1}$ tensor feature maps ${\left\{F_{i,\;l}^{1}\right\}}_{l=1}^{M_{1}}$ after the first convolution layer.

Before inputting the second convolution layer, the edges of ${\left\{F_{i,\;l}^{1}\right\}}_{l=1}^{M_{1}}$ are first zero-complemented so that the tensor feature map has the same size as the initial tensor $Z_{i}$. Then, a similar operation to the first layer is performed on each tensor feature map to obtain $M_{2}$ second-order convolution kernels:

(16) $$W_{g}^{2}=u_{1,g}^{2}\circ u_{2,g}^{2}\circ u_{3,g}^{2}$$

where $g=1,2,\cdots ,M_{2}$. Similarly, convolving each $F_{i,\;l}^{1}$ with each of the $M_{2}$ convolution kernels yields a feature tensor map.

(17) $$G_{i,l,g}^{2}=F_{i,\;l}^{1}*W_{g}^{2}$$

Then the second convolutional layer will eventually output $M_{1}M_{2}$ feature tensors ${\left\{{\left\{G_{i,l,g}^{2}\right\}}_{g=1}^{M_{2}}\right\}}_{l=1}^{M_{1}}$.

**Remark.**  *This section extracts the temporal and spatial features of the video expression sequences based on the 3DPCA and uses this to construct the corresponding 3D convolution kernels for tensor convolution operations, effectively preserving the temporal information of expression changes. However, the feature tensors contain multiple local feature channels, which have some redundancy and are not conducive to the feature representation of the model if directly binarized hash encoding is performed. Therefore, the encoding of the tensor features is improved in the next subsection.*

### 3.2. Feature Encoding Layer Based on the Channel Attention Mechanism

Local channel features at adjacent spatial locations in each tensor feature map output after 3DPCA convolution often have some correlation, due to the overlap of sensory fields. However, in the feature encoding process of a typical PCANet, for each feature map, only weight is usually assigned by contribution and all local features are weighted based on this weight, ignoring the relationship between local channel features and also failing to dynamically adjust the weights of each feature according to the input.

Therefore, this paper addresses the redundancy of local channel features and introduces the SENet to learn adaptive weights for local channel features in each tensor feature to improve the representation capability of the network. The specific steps are as follows.

First, the individual feature tensor maps are still binarized to obtain a feature tensor containing only 0 and 1 elements:

(18) $${\hat{G}}_{i,l,g}^{2}=B\left(G_{i,l,g}^{2}\right)$$

where $B\left(\cdot \right)$ is the binarization function. Omit the lower marker of ${\hat{G}}_{i,l,g}^{2}$ as $G\in R^{H_{1}\times H_{2}\times H_{3}}$, then using each of the three directions of the tensor as a channel dimension yields $G^{\left(1\right)}\in R^{H_{2}\times H_{3}\times H_{1}}$, $G^{\left(2\right)}\in R^{H_{1}\times H_{3}\times H_{2}}$, $G^{\left(3\right)}\in R^{H_{1}\times H_{2}\times H_{3}}$, and enter the SENet one by one to calculate the weights in each of the three directions. First, the first step of the Squeeze operation is performed on $G^{\left(1\right)}={\left\{G_{i}^{\left(1\right)}\right\}}_{i=1}^{H_{1}}$ to obtain a global description of the feature tensor $S^{\left(1\right)}={\left[s_{1},s_{2},\cdots ,s_{H_{1}}\right]}^{T}\in R^{H_{1}}$, where:

(19) $$s_{i}=\frac{1}{H_{2}H_{3}}\sum_{j=1}^{H_{2}}\sum_{k=1}^{H_{3}}G_{i}^{\left(1\right)}\left(j,k\right)$$

After compressing the feature map to obtain a global description of the features, the Excitation operation is then used to calculate the weight matrix:

(20) $$e^{\left(1\right)}=\sigma \left(w_{2}\delta \left(w_{1}S^{\left(1\right)}\right)\right)$$

where $e^{\left(1\right)}\in R^{H_{1}}$, $w_{1}\in R^{\frac{H_{1}}{r}\times H_{1}}$, $w_{2}\in R^{H_{1}\times \frac{H_{1}}{r}}$ are hyperparameters, δ(⋅) denotes the *ReLU* activation function, and σ(⋅) denotes the *Sigmoid* activation function.

Performing the same operation for $G^{\left(2\right)}$ and $G^{\left(3\right)}$, the corresponding weight matrices $e^{\left(2\right)}\in R^{H_{2}}$ and $e^{\left(3\right)}\in R^{H_{3}}$ are obtained. The three weight matrices are then used to weight each element of the feature map tensor with the following weighting formula:

(21) $$\tilde{G}\left(i,j,k\right)=G\left(i,j,k\right)\cdot \frac{e_{i}^{\left(1\right)}+e_{j}^{\left(2\right)}+e_{k}^{\left(3\right)}}{3}$$

The final attention-weighted feature map tensor $\tilde{G}$ is obtained, i.e., ${\tilde{G}}_{i,l,g}^{2}$. Next, the $M_{2}$ feature map tensors are weighted by the degree of contribution to obtain:

(22) $$\Gamma_{i}^{l}=\sum_{g=1}^{M_{2}}2^{g-1}{\tilde{G}}_{i,l,g}^{2}$$

$\Gamma_{i}^{l}$ is then divided into *C* tensor blocks and the histograms of each tensor block are counted to obtain a histogram tandem feature $\tau_{i}^{l}\in R^{2^{M_{2}}C}$ for each feature map $\Gamma_{i}^{l}$. The final cascade of $M_{1}$ histogram features into one feature vector output:

(23) $$v_{i}={\left[\tau_{i}^{1},\tau_{i}^{2},\cdots ,\tau_{i}^{M_{1}}\right]}^{T}\in R^{2^{M_{2}}CM_{1}}$$

The samples can output a final video expression feature matrix after feature extraction using SE-3DPCANet, and then the SVM classifier is used to achieve expression recognition.

**Remark.**  *This subsection uses SENet to learn adaptive weights for local feature channels on the tensor feature map after binarization, reducing feature redundancy. It also avoids the need to weigh all channels based on a single weight during hash coding, increasing the representational power of the model and contributing to subsequent classification accuracy.*

## 4. Case Study

### 4.1. Introduction to the Data Set and Preprocessing

In this paper, three video face expression datasets, CK+ [48], AFEW [49] and CASME II [50], are selected for experimental validation of the algorithm performance.

The CK+ expression dataset consists of a total of 593 video sequences, including 327 tagged expression sequences consisting of seven expressions: anger, contempt, disgust, fear, happiness, sadness, and surprise, with the remaining sequences being neutral expressions. The expressions in each video sequence change from neutral to peak expressions, so 16 frames can be intercepted at appropriate intervals as the expression sequences for this experiment, and the sequences with less than 16 frames are copied from the last frame until 16 frames are made up. All expression frames are then aligned and cropped, with a uniform crop size of 64×64; this gives the tensor size of each video sample as 64×64×16. Finally, the processed expression sequences in each class were divided 3:1 into the training and test sets.

The AFEW dataset is derived from film footage and is more realistic and challenging than the laboratory’s posed expressions. The parts of the film containing more expressions were selected for the interception, and a total of 1809 video expression sequences were obtained, containing seven expression labels: anger, disgust, fear, happiness, sadness, surprise, and neutral. For each video sequence, 16 frames at regular intervals were selected as the experimental sample sequence and then aligned and cropped according to the same criteria.

The CASME II micro-expressions dataset consists of 255 micro-expression video sequences from 26 filmmakers, containing a total of five basic expressions: boredom, happiness, surprise, sadness, and fear, as well as other expressions. Each video sequence changes from a neutral expression to a peak expression and finally back to a neutral expression. Therefore, for each video sequence, 16 frames are intercepted at regular intervals from the beginning of the neutral expression to the end of the peak, and the interval is used as a sample of the expression sequence. The same alignment and cropping operations are then performed with the CK+ dataset.

### 4.2. Experiment 1: Selection of Model Parameters

This subsection conducts experiments on the selection of parameters for the proposed SE-3DPCANet model. The optimal parameter values for the network can be selected by a combinatorial comparison. Uniformly set the number of filters to $M_{1}=M_{2}=8$, the sampling block plane sliding size $k_{1}\times k_{2}$ is chosen as 9×9, 11×11, 13×13 separately, sample block sliding frame number $k_{3}$ increased from 8 to 16, the histogram window size is 7×7×7, and the corresponding overlap rate was set to 0.5. The same trained SVM classifier was used for expression classification recognition.

**Figure 5:**
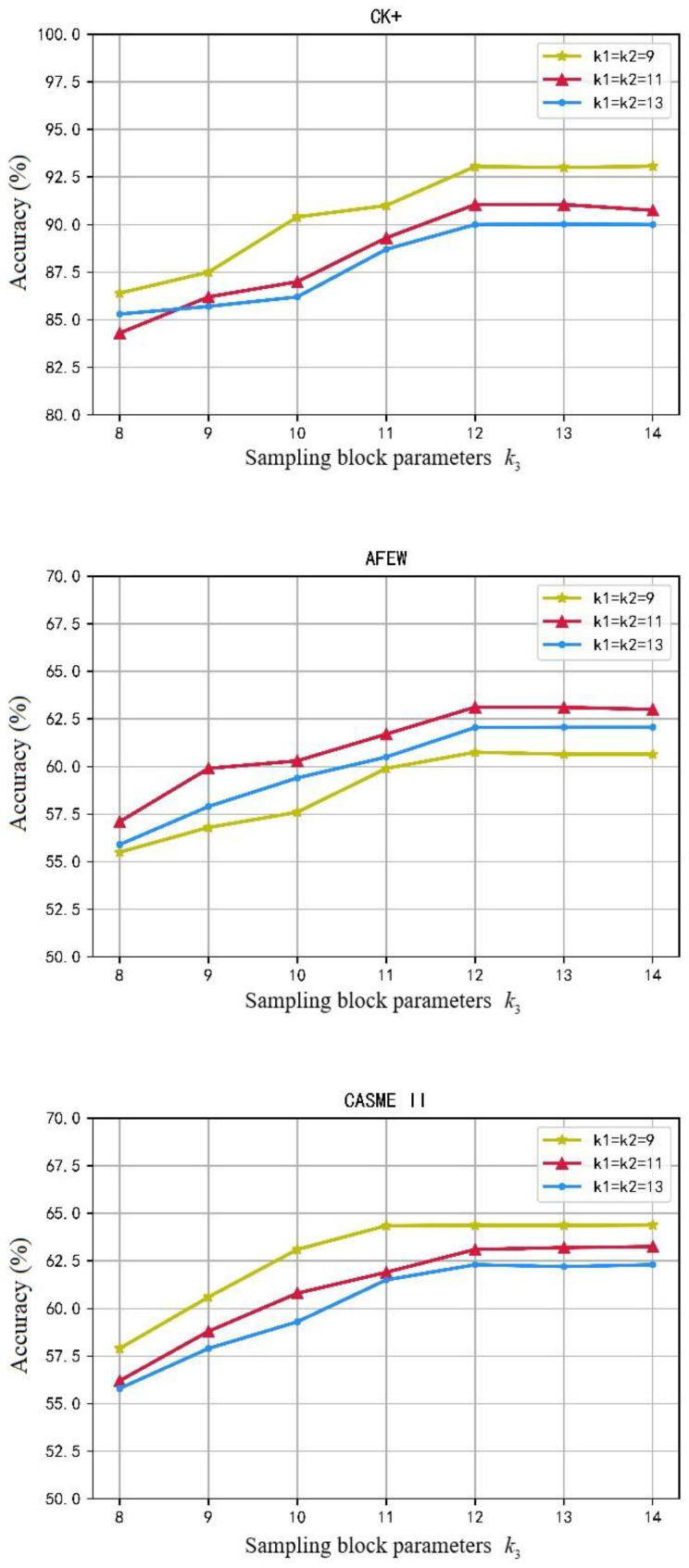
Recognition rate curve for SE-3DPCANet with different parameters.

The SE-3DPCANet recognition rate curves for different parameters are shown in Figure 5. As can be seen from the parameter curves in Figure 5, the recognition accuracy of the model increases with the increase of the sampling size in the spatial dimension and the sampling size in the temporal dimension under all three datasets, and eventually stabilizes. The optimal combination of sampling block parameters for the CK+ and CASME II datasets is $k_{1}=k_{2}=9$, $k_{3}=12$. By comparison, the optimal combination of sampling block parameters for the AFEW dataset is $k_{1}=k_{2}=11$, $k_{3}=12$.

### 4.3. Experiment 2: Algorithm Performance Comparison and Analysis

**Figure 6:**
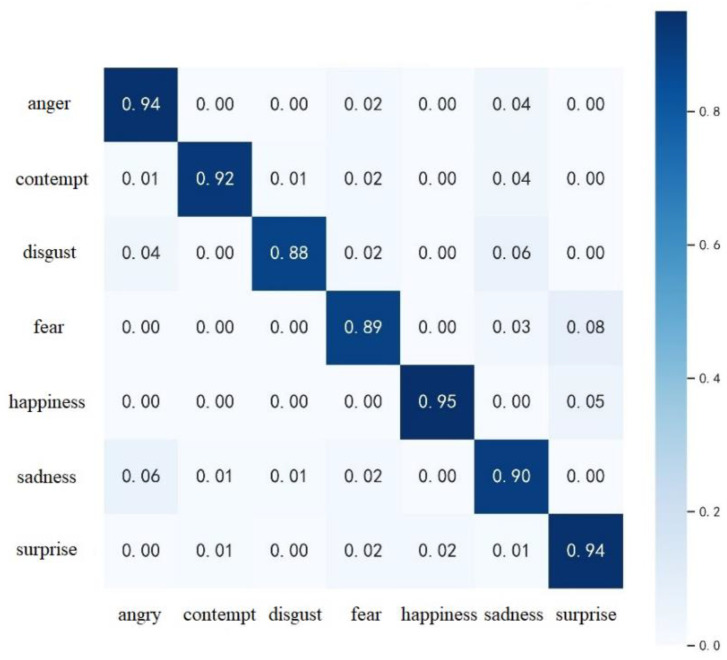
Confusion matrix for CK+ dataset.

This subsection tests the performance of SE-3DPCANet on three datasets: CK+, AFEW and CASME II, respectively. The confusion matrix for SE-3DPCANet on the CK+ dataset was first calculated, as shown in Figure 6. The confusion matrix provides a visual representation of the classification accuracy of the model, which indicates the proportion of each type of expression predicted by the model in the actual sample of each type of expression, as a way to assess the classification performance of the SE-3DPCANet model.

According to Figure 6, it can be seen that the recognition accuracy of all types of expressions in the CK+ dataset is relatively high. Thanks to the fact that the facial expressions in the CK+ dataset were all filmed in the laboratory, the video images are of better quality and the expression movements are in place, making them easier to distinguish and recognize. Among the seven expressions in the CK+ dataset, the recognition accuracy of happiness, surprise, and anger is relatively high. Of these, the happiness expression is the highest, as it is characterized by more intense emotions and larger movements. In addition to this, happiness has an advantage in terms of sample size. However, the recognition rate of disgust, fear, and sadness expressions is low compared to other expressions. The confusion matrix shows that disgust is easily misidentified as sadness; fear is easily confused with surprise; and a small number of sadness expressions are recognized as anger. The main reasons for this are the presence of similar facial movement features in some expressions and the unevenness of the samples, which affects the recognition rate.

The SE-3DPCANet algorithm proposed in this paper is then compared with existing expression recognition algorithms 3D-CNN, 3D Inception-ResNet, Spatio-temporal manifold, as well as PCANet, KPCANet-LDA, 3D-PCANet. A comparison of the recognition performance of each algorithm on the CK+ dataset is shown in Table 1.

Table 1 shows that the recognition rate of the SE-3DPCANet model proposed in this paper is 93.15% on the CK+ dataset, which is higher than most of the algorithms in the table. The comparison between SE-3DPCANet and 3DPCANet illustrates that the introduction of the SENet facilitates the extraction of more effective features and maintains a shorter training time while improving the recognition rate. Compared with the 3D-ResNet algorithm, the training time of the proposed method is less than 1/21 of that of 3D-ResNet, although the correct recognition rate of the proposed method is reduced by 1.05%. The proposed method is more suitable for realistic needs, as it greatly reduces training costs while significantly preserving the accuracy of the model.

**Figure 7:**
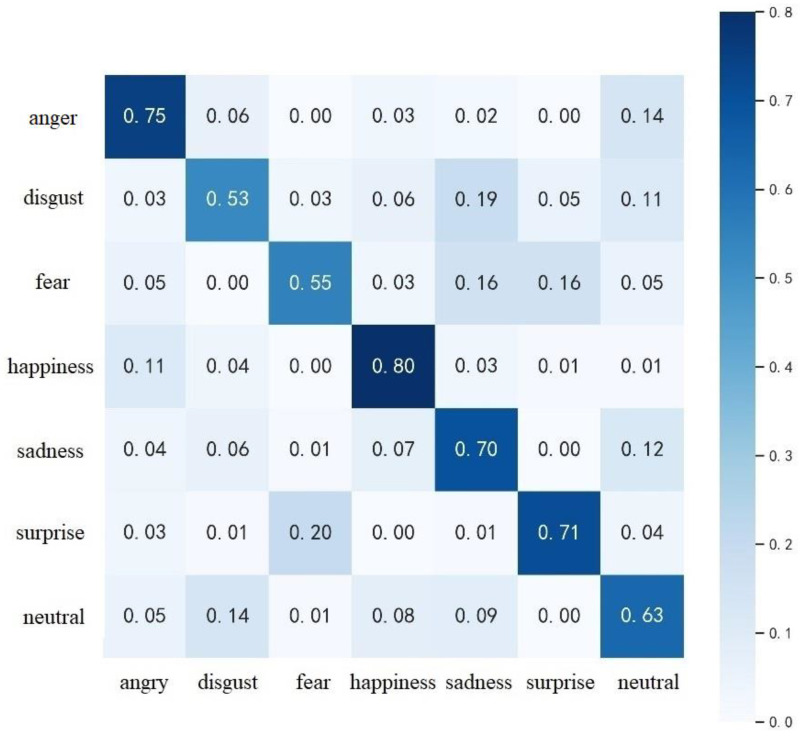
Confusion matrix for the AFEW dataset.

**Figure 8:**
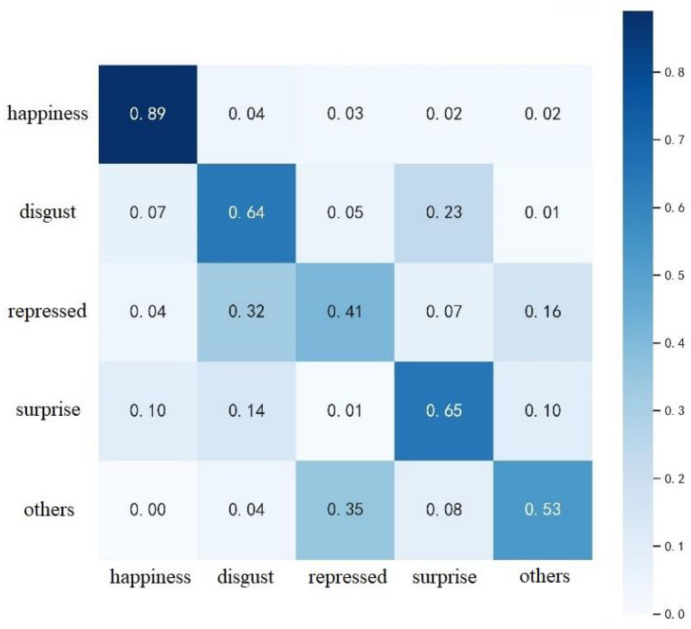
Confusion matrix for the CASME II dataset.

The confusion matrix for SE-3DPCANet on the AFEW dataset was calculated using the same method as shown in Figure 7. Compared with Figure 8, it can be seen that the overall recognition rate of the proposed algorithm on AFEW is smaller than that of CK+, which is mainly affected by the lack of resolution of the face video image and the fullness of the expression action, and there is also a certain gap in the number and quality of samples for each type of expression. The proposed algorithm achieves the highest recognition rate of 80% for happiness and more than 70% for anger, surprise and sadness, but the recognition rate for disgust and fear expressions is low. It can be seen from Figure 7 that some of the disgust expressions were identified as sadness and some of the fear expressions were identified as sadness and surprise. Both disgust and sadness are negative emotions, which inherently share two very similar sets of facial expressions. In addition, there are many similar expression frames in the video sequences of disgust and sadness expressions, resulting in greater misclassification of disgust and sadness on the AGEW dataset. When the negative stimulus source exceeds the test subject’s psychological expectations, the emotion of fear arises; when a bad result occurs, the test subject’s emotion changes from fear to sadness. Thus, there was a strong correlation between sadness and fear, resulting in some of the fearful expression samples being misclassified as sadness.

The SE-3DPCANet algorithm was then compared with 3D-CNN, VGG-LSTM, VGG-LSTM-Attention, 3DCNN-Attention, PCANet, and 3D-PCANet on the AFEW dataset. A comparison of the performance of the various algorithms on the AFEW dataset is shown in Table 2.

Comparing the recognition rates and training times of the algorithms in Table 2, it is clear that the SE-3DPCANet can also achieve recognition rates similar to or even higher than the deep convolutional network on the AFEW dataset, while having a lower training time. Compared with the highest recognition rate of 3DCNN-Attention, the recognition rate of the proposed method is only 0.45% lower; however, the training time required for 3DCNN-Attention is more than 1 day, while the training time required for the proposed method is only 3 h. The extremely long training time of 3DCNN-Attention is unacceptable for subsequent sample replenishment, parameter changes and model updates. The proposed method significantly reduces the time required for model training while substantially ensuring model accuracy compared to other methods. It is well-illustrated that the use of SE-3DPCANet can effectively extract feature information on expression sequences, especially preserving the temporal correlation between expression action frames.

The final calculation of the confusion matrix for SE-3DPCANet on the CASME II dataset is shown in Figure 8. As can be seen in Figure 8, the overall expression recognition rates of the algorithms proposed in this paper are all low on CASME II. The best recognition is achieved in happiness, with an accuracy of 89%, which is due to the fact that facial changes are more obvious when a subject is happy, which facilitates recognition. The lowest recognition rate was for repressed, which is because repressed faces have smaller movements and less distinctive features, making it easy to misidentify expressions. The recognition rate for all remaining expressions is low, with disgust and surprise easily confused and some of the other expressions being incorrectly categorized as depressed.

The SE-3DPCANet algorithm was then compared with the common expression recognition methods LBP-TOP, EVM+HIGO, CNN-LSTM, PCANet, and 3D-PCANet on the CASME II dataset. A comparison of the performance of each algorithm is shown in Table 3.

Table 3 shows that SE-3DPCANet also achieves good recognition results on the CASME II dataset, and can achieve a final recognition rate of 64.17%. The recognition rate is 3.08% lower than that of the EVM + HIGO algorithm, but, with its lightweight network structure, it greatly reduces the time cost in model training, which is less than 1/17th of the training time required by EVM + HIGO. Most of the remaining methods not only have a longer training time, but the model accuracy is not as excellent as the method proposed in this paper. Although the training time is shorter using only PCANet models, the significantly lower model accuracy is not acceptable in practical situations.

## 5. Conclusions

In this paper, we propose a video sequence face expression recognition method based on Squeeze-and-Excitation and 3DPCA Network. The model input samples are video expression sequences, and a tensor convolution kernel is constructed in the convolution layer, using 3DPCA to extract the dynamic expression features of the 3D video sequences; then, for the redundancy of local channel features, the channel attention mechanism module SENet is introduced in the feature encoding layer, to automatically learn the weights of local features in the tensor features to further improve the recognition accuracy. Experimental validation was conducted on three datasets: CK+, AFEW, and CASME II, respectively. The recognition accuracy of the model was evaluated by calculating the confusion matrix for each type of expression and comparison with other common expression recognition algorithms for analysis. Experimental results show that the SE-3DPCANet algorithm proposed in this paper can effectively recognize various types of expressions and achieve close to or even higher correct recognition rates compared to most expression recognition methods. The proposed method has great advantages in terms of training time, and the overall performance of the model is superior.

We list the benefits and drawbacks as follows:

**Benefits:**

(1) The facial expression features extracted by 3DPCANet greatly reduce the redundancy of the original information while retaining the key information.

(2) SENet increases the representation capability of the model and, in combination with 3DPCANet, greatly reduces the time cost of model training while ensuring the accuracy of the model as much as possible.

**Drawbacks:**

(1) While the proposed method substantially reduces the time required for training, the model accuracy is also slightly reduced compared to deep neural networks. How to maintain or even surpass the accuracy of the original model while reducing the training time of the model, and significantly improve the comprehensive performance of the model, is a future research direction.

(2) How to apply the proposed method to micro-expression recognition and extract the small difference in features of human facial expression is a future research direction.

## Figures and Tables

**Table 1:** Performance comparison of different algorithms on the CK+ dataset.

Algorithms	Recognition Rate (%)	Training Time (hours)
3D-CNN [44]	92.43	>24
3D Inception-ResNet [44]	93.21	>12
Spatio-temporal manifold [51]	94.20	10
PCANet	88.65	0.381
KPCANet-LDA [52]	91.32	-
3D-PCANet	92.67	0.524
SE-3DPCANet	93.15	0.568

**Table 2:** Performance comparison of different algorithms on the AFEW dataset.

Algorithms	Recognition Rate (%)	Training Time (hours)
3D-CNN [44]	35.23	>24
VGG-LSTM [53]	47.40	>12
VGG-LSTM-Attention [42]	61.11	16
3DCNN-Attention [54]	63.24	>24
PCANet	32.44	2.17
3D-PCANet	61.86	2.84
SE-3DPCANet	62.79	3.01

**Table 3:** Performance comparison of different algorithms on the CASME II dataset.

Algorithms	Recognition Rate (%)	Training Time (hours)
LBP-TOP [55]	46.33	11
EVM + HIGO [56]	67.25	9
CNN-LSTM [57]	61.04	>12
PCANet	52.19	0.352
3D-PCANet	62.53	0.485
SE-3DPCANet	64.17	0.519

## Data Availability

No new data were created or analyzed in this study. Data sharing is not applicable to this article.
